# Astragaloside IV attenuates renal tubule injury in DKD rats via suppression of CD36-mediated NLRP3 inflammasome activation

**DOI:** 10.3389/fphar.2024.1285797

**Published:** 2024-03-20

**Authors:** Xianhong Li, Xin Dong, Liangyou Zhang, Shu Zhang, Weiying Huang, Chao Wang, Zhihao Huo, Xin Li, Xiwen Zhang, Xiaotong Jia, Gangyi Chen, Bin Kuang

**Affiliations:** ^1^ The First Affiliated Hospital of Guangzhou University of Chinese Medicine, Guangzhou, China; ^2^ Dongguan Hospital of Traditional Chinese Medicine, Dongguan, China; ^3^ Shenzhen Luohu District Traditional Chinese Medical Hospital, Shenzhen, China; ^4^ Guangzhou University of Chinese Medicine, Guangzhou, China

**Keywords:** diabetic kidney disease, Astragaloside IV, PA-induced HK-2 cells, CD36, NLRP3 inflammasome

## Abstract

**Background:**

In recent years, diabetic kidney disease (DKD) has emerged as a prominent factor contributing to end-stage renal disease. Tubulointerstitial inflammation and lipid accumulation have been identified as key factors in the development of DKD. Earlier research indicated that Astragaloside IV (AS-IV) reduces inflammation and oxidative stress, controls lipid accumulation, and provides protection to the kidneys. Nevertheless, the mechanisms responsible for its protective effects against DKD have not yet been completely elucidated.

**Purpose:**

The primary objective of this research was to examine the protective properties of AS-IV against DKD and investigate the underlying mechanism, which involves CD36, reactive oxygen species (ROS), NLR family pyrin domain containing 3 (NLRP3), and interleukin-1β (IL-1β).

**Methods:**

The DKD rat model was created by administering streptozotocin along with a high-fat diet. Subsequently, the DKD rats and palmitic acid (PA)-induced HK-2 cells were treated with AS-IV. Atorvastatin was used as the positive control. To assess the therapeutic effects of AS-IV on DKD, various tests including blood sugar levels, the lipid profile, renal function, and histopathological examinations were conducted. The levels of CD36, ROS, NLRP3, Caspase-1, and IL-1β were detected using western blot analysis, PCR, and flow cytometry. Furthermore, adenovirus-mediated CD36 overexpression was applied to explore the underlying mechanisms through *in vitro* experiments.

**Results:**

*In vivo* experiments demonstrated that AS-IV significantly reduced hyperglycemia, dyslipidemia, urinary albumin excretion, and serum creatinine levels in DKD rats. Additionally, it improved renal structural abnormalities and suppressed the expression of CD36, NLRP3, IL-1β, TNF-α, and MCP-1. *In vitro* experiments showed that AS-IV decreased CD36 expression, lipid accumulation, and lipid ROS production while inhibiting NLRP3 activation and IL-1β secretion in PA-induced HK-2 cells.

**Conclusion:**

AS-IV alleviated renal tubule interstitial inflammation and tubule epithelial cell apoptosis in DKD rats by inhibiting CD36-mediated lipid accumulation and NLRP3 inflammasome activation.

## 1 Introduction

Diabetic kidney disease (DKD), a significant microvascular complication of diabetes mellitus (DM), is the primary cause of end-stage renal disease (ESRD) globally (Chan and Tang, 2013). Previous studies have primarily focused on glomerular damage, but emerging research indicates that DKD is not only caused by glomerular damage, but also by complex interactions among glomeruli, tubules, renal interstitium, and renal vascular components (Kanwar et al., 2011). In comparison to glomerular injury, renal tubule and tubulointerstitial injury exert a more significant impact on renal function and offer greater predictive value for DKD progression (Vallon, 2011). Nevertheless, the specific mechanism behind these observations is still not understood.

Recent studies have demonstrated the prominent role of inflammatory mechanisms, specifically the cytokine interleukin-1β (IL-1β) and the NLRP3 inflammasome, in promoting DKD (Navarro-González et al., 2011). The NLRP3 inflammasome is a protein complex consisting of NOD-like receptor protein 3 (NLRP3), apoptosis-related speck protein (ASC), and cysteine aspartate-1 precursor that activates IL-1β family cytokines (Qiu and Tang, 2016). An increasing body of evidence suggests that activation of the NLRP3 inflammasome plays a pivotal role in the progression of DKD. According to Wu *et al.* (Wu et al., 2018), suppression of the NLRP3 inflammasome decreases inflammation and fibrosis in renal tissue in DKD. Furthermore, activation of the NLRP3 inflammasome is also associated with increased levels of oxidized low-density lipoprotein (ox-LDL), free fatty acids (FFAs), and lipid accumulation, in addition to hyperglycemia and oxidative stress (Duewell et al., 2010). There is a growing body of evidence indicating that inflammation, fibrosis, and disease progression may be associated with dyslipidemia in patients with DKD (Herman-Edelstein et al., 2014; Stadler et al., 2015). CD36-mediated lipid accumulation may be one potential pathogenic factor of obesity-related glomerulopathy (ORG) and podocyte injury through inflammatory responses and activation of the NLRP3 inflammasome (Yang et al., 2018; Zhao et al., 2019). Another study suggests that CD36 mediates NLRP3 inflammasome activation via suppressing mitochondrial fatty acid oxidation (FAO) and stimulating mitochondrial reactive oxygen species (mtROS) production in DKD (Hou et al., 2021).

CD36, which is recognized as a protein responsible for transporting fatty acids, is a scavenger receptor B that displays a wide array of sites for recognizing ligands (Yokoi and Yanagita, 2016). It plays a crucial role in lipid deposition, inflammatory signaling pathways, energy reprogramming, apoptosis, and renal interstitial fibrosis. CD36 is predominantly found in tubular epithelial cells, podocytes, and mesangial cells within the kidney. It enhances lipid absorption, triggers cell death, and stimulates the production of ROS (Hua et al., 2015; Yang et al., 2017). Patients with chronic kidney disease (CKD), especially those with diabetic kidney disease, exhibit significant expression of CD36. We suggest that the accumulation of lipids through CD36 and activation of the NLRP3 inflammasome may play a role in the potential development of renal tubule injury in DKD.

Astragaloside IV (AS-IV), a bioactive substance derived from Radix Astragali, demonstrates remarkable abilities in reducing oxidative stress and inflammation and providing protection to the kidneys, as well as modulating the immune system (Zhou et al., 2017; Chen et al., 2018). AS-IV effectively suppresses oxidative stress and fibrosis induced by palmitic acid (PA) through the downregulation of CD36 in glomerular mesangial cells (Su et al., 2019). In our prior investigation, it was shown that AS-IV alleviates lipid accumulation, decreases damage to cardiomyocytes, and improves myocardial dysfunction in DCM rats by inhibiting CD36-mediated ferroptosis (Li et al., 2023).

In the present study, we explored the impact of AS-IV on the PA-induced inflammation of renal tubular epithelial cells. We also examined the protective effects of AS-IV in a DKD rat model induced by streptozotocin along with a high-fat diet. Our findings demonstrate that AS-IV regulates lipid accumulation, reduces the production of ROS, and inhibits the activation of NLRP3 inflammatory bodies by acting on CD36. As a result, it effectively suppresses the release of the inflammatory factor IL-1β in renal tubular epithelial cells. This leads to reduced renal tubulointerstitial inflammation and delayed progression of DKD. Moreover, the impacts of AS-IV on FFA-induced damage to the renal tubules and the underlying mechanisms were examined to elucidate the correlation between CD36 and the protective properties of AS-IV in HK-2 cells exposed to PA, as well as in a DKD rat model induced by streptozotocin along with a high-fat diet.

## 2 Materials and methods

### 2.1 Chemicals and reagents

The human renal proximal tubular HK-2 cell line (Cat. No. CL-0109) was acquired from Procell Life Science & Technology Co., Ltd. (Wuhan, China). PA (C16:0) (Cat. No. P5585) and streptozotocin (Cat. No. S0130) were obtained from Sigma-Aldrich (St. Louis, MO, United States). Atorvastatin was purchased from The First Affiliated Hospital of Guangzhou University of Chinese Medicine. Anti-NLRP3 (Cat. No. 27458-1-AP), anti-CD36 (Cat. No. 18836-1-AP), anti-ASC (Cat. No. 10500-1-AP), anti-cleaved-caspase-1 (Cat. No. 81482-1-RR), anti-cleaved-IL-1β (Cat. No. AF4006), Anti-Cleaved-GSDMD (Cat. No. 36425), Anti-GSDMD (Cat. No. 39757), Anti-KIM-1 (Cat. No. ab78494), Anti-L-FABP (Cat. No. 13626-1-AP), Anti-NGAL (Cat. No. 26991-1-AP), and anti-α-tubulin (Cat. No. 11224-1-AP) were purchased from Proteintech Group, Inc. (Wuhan, China). RIPA lysis buffer (Cat. No. P0013D), the BCA kit (Cat. No. 010719190225), and the SDS-PAGE gel preparation kit (Cat. No. 120120201209) were purchased from Beyotime Biotechnology (Shanghai, China). The Oil Red O staining kit (Cat. No. G1262) was obtained from Solarbio Science & Technology (Beijing, China). AS-IV (purity > 98% as determined by HPLC) was purchased from Nanjing Chunqiu Bioengineering Co., Ltd. (Nanjing, China).

### 2.2 Animal grouping and DKD model establishment

Male Sprague-Dawley rats (170 ± 10 g) were acquired from the Experimental Animal Center at Guangzhou University of Chinese Medicine. After 1 week of adaptive feeding, the rats were randomly divided into two groups: the control group (normal diet, *n* = 6) and the high-fat diet (HFD) group (*n* = 30). The experimental method refers to our previous study (Li et al., 2023). Rats were considered diabetic after three consecutive days of fasting blood glucose levels exceeding 16.7 mmol/L. Rats in the HFD group were randomly assigned to one of five groups (*n* = 6 rats per group): the DKD model group, the positive control group receiving atorvastatin treatment (10 mg/kg, administered via gavage), and the AS-IV treatment groups (20, 40, and 80 mg/kg/day). Throughout the experiment, body weight was evaluated on a weekly basis. Additionally, fasting blood glucose (FBG) was tested every 4 weeks after a 6-h period of fasting. 24-h urine samples were collected using individual metabolic cages. All rats were sedated and sacrificed after 12 weeks of treatment. Serum was collected for biochemical analysis. For pathological analysis, the left kidneys were immersed in paraffin, while the right kidneys were preserved at −80°C. Approval for this study was granted by the Animal Ethics Committee of the First Affiliated Hospital of Guangzhou University of Chinese Medicine (approval number TCMF1-2019009).

### 2.3 Serum and urine biochemistry

The rat microalbuminuria ELISA Kit (Cat. No. CSB-E12991r) was utilized to determine the urinary albumin concentration. The urinary creatinine level was measured using the Creatinine Assay Kit (Cat. No. C011-2-1, Nanjing Jiancheng Bioengineering Institute) according to the manufacturer’s instructions. The levels of serum triacylglycerol (TG), total cholesterol (TC), low-density lipoprotein–cholesterol (LDL-C), blood urea nitrogen (BUN), and serum creatinine (Scr) were measured using a Roche automatic biochemistry analyzer (Model No. Cobas c702, Roche, Japan) at the Laboratory Department of the First Affiliated Hospital of Guangzhou University of Traditional Chinese Medicine.

### 2.4 Renal pathology and immunohistochemistry

To evaluate the histology of the kidneys, the kidney tissues were first treated with 4% paraformaldehyde for 48 h, followed by dehydration and embedding in paraffin. Thin tissue sections (4 μm) were prepared for staining with hematoxylin and eosin (H&E), periodic acid–Schiff (PAS), and Masson’s trichrome. To perform immunofluorescence staining, the kidney sections were treated with anti-NLRP3 as per the guidelines provided by the manufacturer. Staining analysis was conducted using light microscopy by two separate blinded observers. The collected images were assessed using ImageJ 1.8.0 (National Institutes of Health, United States).

### 2.5 Cell culture and transfection

HK-2 cells were cultured in DMEM/F12 medium (Cat. No. BL305A, biosharp, China) supplemented with 10% fetal bovine serum (Cat. No. 10100147, Gibco, United States), 100 μg/mL streptomycin, and 100 U/mL penicillin (Cat. No. 10100147, Gibco, United States) in a 5% CO_2_ incubator at 37°C. Cells were divided into five groups: (1) the control group (cells treated with 1% BSA), (2) the PA group (cells treated with 400 mM PA), and (3–5) the PA + AS-IV groups (20, 40, and 80 µM AS-IV). To investigate the impact of AS-IV on CD36 expression in PA-induced HK-2 cells, we enhanced the expression of CD36 in HK-2 cells by utilizing a lentiviral expression vector. The CD36 overexpression adenovirus was acquired from Genechem Co., Ltd. (Shanghai, China). HK-2 cells in the 80 μM AS-IV group were transfected with the CD36 overexpression plasmid. Western blot analysis and PCR were used to determine the expression levels of CD36 in HK-2 cells.

### 2.6 Cytotoxicity test

We assessed the toxic effects of AS-IV by employing the Cell Counting Kit-8 (CCK-8, MIKX, Shenzhen, China). HK-2 cells were cultured in 96-well plates at a density of 1 × 10^4^ cells/mL in 100 μL of culture medium for 48 h. Different concentrations of AS-IV (0, 20, 40, 80, and 120 μM) were added, with or without PA (400 mM), in 10 μL. After incubating for 2 h, 10 μL of CCK-8 reagent was added to each well, and the cells were incubated again. The absorbance at 450 nm was determined with a microplate reader (Thermo Fisher Scientific, Waltham, United States). The experiments were carried out in triplicate.

### 2.7 Oil Red O staining

HK-2 cells were stained with Oil Red O according to the manufacturer’s instructions, following the experimental steps described in our previous study (Li et al., 2023). HK-2 cells were seeded into six-well dishes and incubated, and the initial medium was replaced. The cells were rinsed two times using PBS, treated with Oil Red fixative for 30 min, and finally washed two times with distilled water. Afterwards, the cells were rinsed with 60% isopropanol for 20–30 s, which was then removed; subsequently, Oil Red O staining solution was added. After 20 min, the Oil Red O solution was disposed of, and the cells were washed using 60% isopropanol for 20–30 s until the interstitial space turned transparent. The dishes were additionally rinsed five more times using distilled water until all excess stain was removed. Afterwards, Oil Red O buffer was introduced and removed within 1 min. In the end, purified water was included to ensure the cells were fully covered for examination using a fluorescence microscope.

### 2.8 Quantitative real-time PCR (RT-PCR) analysis

Total RNA was extracted from both rat kidney tissues and HK-2 cells using TRIzol reagent (Ca. No. QP020, MRC, United States) and then converted to cDNA using the FastKing RT Kit (Ca. No. KR116, TIANGEN, China) following the manufacturer’s instructions. A CFX Connect Real-Time PCR System (Bio-Rad, United States) was utilized for quantitative RT-PCR analyses. Primer sequences are presented in Table 1.

**TABLE 1:** Primer sequences.

Gene	Forward sequence	Reverse sequence
CD36	TCG​GAT​GGC​TAG​CTG​ATT​ACT​T	CCT​CGT​GCA​GCA​GAA​TCA​AG
NLRP3	TTG​TTC​TCT​GCA​TGC​CGT​ATC​T	AGG​GTA​CCC​CAT​AGA​CTG​GC
ACS	TTT​GTG​GAC​CAA​CAC​AGG​CA	GTT​GGT​GGT​CTC​TGC​ACG​AA
Caspase-1	GAC​CGA​GTG​GTT​CCC​TCA​AG	GAC​GTG​TAC​GAG​TGG​GTG​TT
CPT-1	CCT​ACC​ACG​GCT​GGA​TGT​TT	TAC​AAC​ATG​GGC​TTC​CGA​CC
PPAR-α	GGC​TCT​GAA​CAT​TGG​CGT​TC	CAA​GGG​GAC​AAC​CAG​AGG​AC
MCP1	AGC​CAA​CTC​TCA​CTG​AAG​CC	AAC​TGT​GAA​CAA​CAG​GCC​CA
TNF-α	ACT​GAA​CTT​CGG​GGT​GAT​CG	GCT​TGG​TGG​TTT​GCT​ACG​AC
IL-6	AGA​GAC​TTC​CAG​CCA​GTT​GC	AGT​CTC​CTC​TCC​GGA​CTT​GT
IL-1β	GCA​CAG​TTC​CCC​AAC​TGG​TA	ACA​CGG​GTT​CCA​TGG​TGA​AG
HIF-1a	TAT​GAG​CCA​GAA​GAA​CTT​TTA​GGC	CAC​CTC​TTT​TGG​CAA​GCA​TCC​TG

### 2.9 Western blot analysis

Total protein was extracted from HK-2 cells or rat renal tissues using RIPA lysis buffer (Cat. No. P0013B, Beyotime, China). The protein concentration was determined using a BCA protein assay kit (Cat. No. P0010S, Beyotime, China) according to the provided instructions. The protein samples were mixed with 5× loading buffer (Cat. No. FD002, Fdbio science, China) and boiled for 5 min. Proteins were separated by SDS-PAGE and then transferred to membranes, which were incubated at room temperature in 5% skimmed milk for 1 h and subsequently exposed to primary antibodies (anti-CD36, anti-NLRP3, anti-cleaved-IL-1β, anti-cleaved-Caspase-1, anti-ASC, anti-GSDMD, anti-cleaved-GSDMD, anti-KIM-1, anti-L-FABP, anti-NGAL, and anti-α-tubulin) overnight at 4°C. Afterward, the membranes were washed, incubated with goat anti-rabbit for 1 h at ambient temperature, and washed again. Enhanced chemiluminescence (ECL, Ca. No. WBKLS0500, Millipore, United States) was performed using Clarity Max™ Western ECL Substrate (Ca. No.1705062, Bio-Rad, United States). The western blots were analyzed with ImageJ software (NIH, MD, United States).

### 2.10 ROS detection

HK-2 cells were seeded into six-well dishes, incubated, washed, trypsinized, suspended in PBS, incubated with MitoSox Red (Cat. No.M36008, Thermo Fisher Scientific Inc., United States) at 37°C for 30 min, washed three times with PBS, and centrifuged. The supernatant was discarded, and flow cytometry analysis was conducted using a FACSCalibur flow cytometer (BD, United States).

### 2.11 Statistical analysis

All data are expressed as mean ± standard deviation. Statistical analysis was performed using GraphPad Prism 9.5.1 software and SPSS 26.0 software. One-way analysis of variance (ANOVA) and Tukey’s multiple comparison test were used for comparisons among all groups. The *t*-test was used for pairwise comparisons. Differences were considered statistically significant if *p* < 0.05.

## 3 Results

### 3.1 Effects of AS-IV on body weight and glycolipid metabolism in DKD rats

**FIGURE 1:**
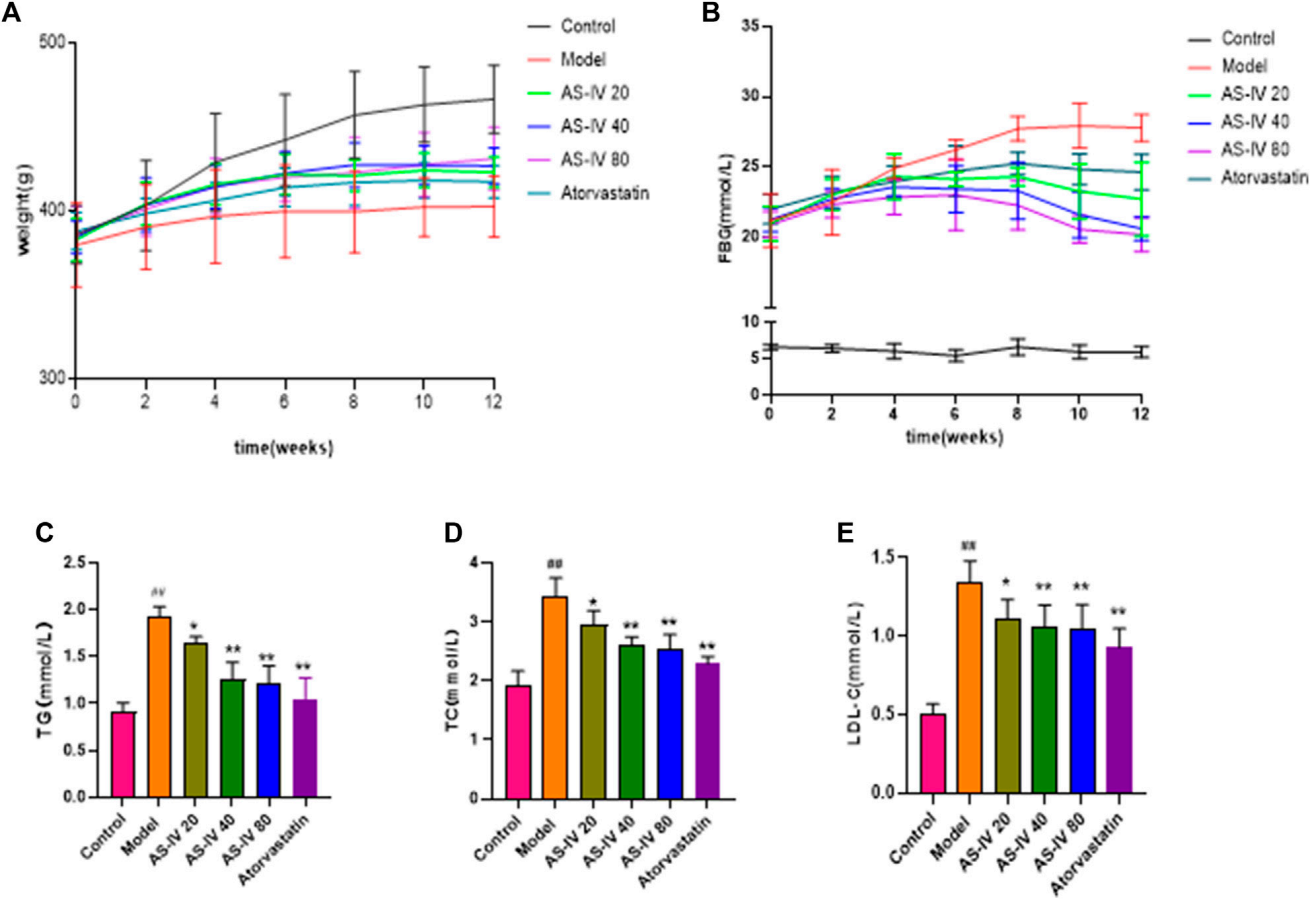
Effects of AS-IV on body weight, FBG, and serum lipid profiles in different groups after 12 weeks of administration. **(A)** Weight, **(B)** FBG, **(C)** TG, **(D)** TC, **(E)** LDL-C. All data are presented as the mean ± SD (*n* = 6). ^##^ *p* < 0.01 vs. Control; ^*^ *p* < 0.05 and ^**^ *p* < 0.01 vs. Model.

Initially, there were no obvious disparities among the six groups in body weight (Figure 1A). However, by week 12, the control group had a significantly higher body weight than the DKD rats (*p* < 0.05). Although the AS-IV treatment group exhibited a consistent rise in weight over the initial 6 weeks compared to the model group, the body weights of all DKD groups remained almost unchanged thereafter.

As shown in Figure 1B, the FBG levels in DKD rats were considerably elevated compared to the control group (*p* < 0.01). However, after 8 weeks of AS-IV administration, a significant decrease in FBG levels was observed in comparison to the model group (*p* < 0.05).

Compared to the control group, the levels of TG, TC, and LDL-C showed a significant increase in DKD rats (Figures 1C–E, *p* < 0.01). However, upon administering AS-IV at doses of 40 and 80 µM for a duration of 12 weeks, a substantial decrease in their levels was observed (*p* < 0.01).

### 3.2 AS-IV treatment protects renal function in DKD rats

**FIGURE 2:**
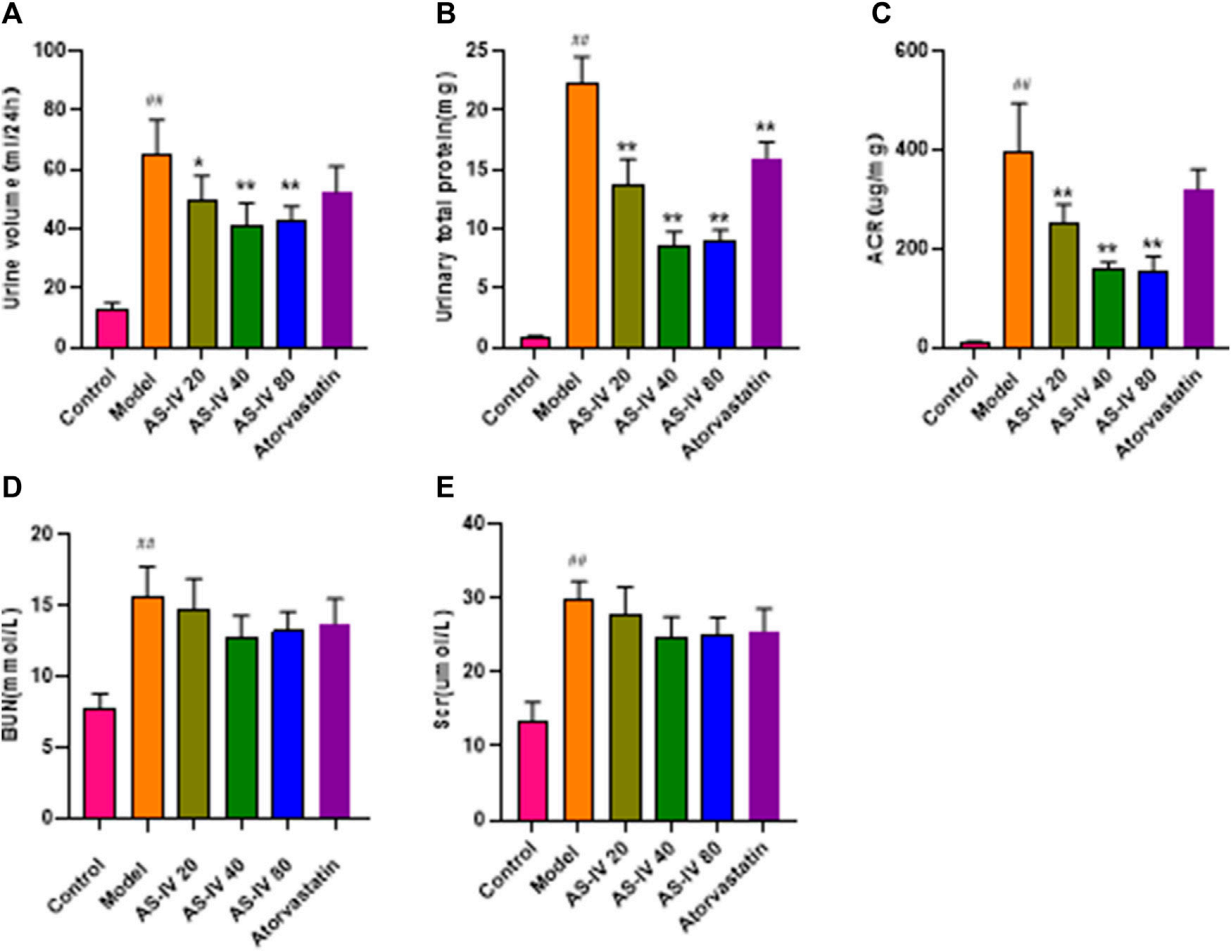
AS-IV improves renal function in different groups after 12 weeks of administration. **(A)** Urine volume, **(B)** Urinary total protein, **(C)** ACR, **(D)** BUN, **(E)** Scr. All data are presented as the mean ± SD (*n* = 6). ^##^ *p* < 0.01 vs. Control; ^*^ *p* < 0.05 and ^**^ *p* < 0.01 vs. Model.

As shown in Figures 2A–E, DKD rats had elevated levels of 24-h urine volume, urinary total protein, albumin-to-creatinine ratio (ACR), BUN, and Scr compared to the control group. Nevertheless, administration of AS-IV led to a decrease in 24-h urine volume, urinary total protein, and ACR in DKD rats (Figures 2A–C, *p* < 0.05). The BUN and Scr levels showed a decline after AS-IV treatment in comparison to the model group, but the difference was not statistically significant (Figures 2D, E, *p* > 0.05).

### 3.3 AS-IV alleviates renal tubular injury, tubulointerstitial inflammation, and NLRP3 in DKD rats

**FIGURE 3:**
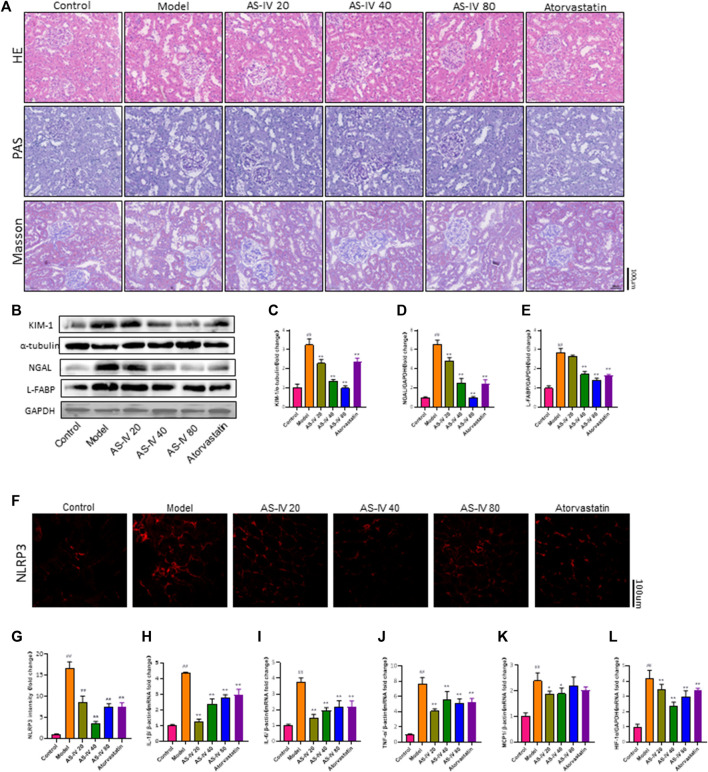
AS-IV ameliorates renal tubular injury in DKD rats. **(A)** Representative images of the pathological structure of the kidneys after H&E, PAS, and Masson’s trichrome staining (bar = 100 μm; magnification, ×200). **(B)** Western blot analysis of KIM-1, NGAL, and L-FABP. **(C–E)** Protein levels of KIM-1 after normalization to α-tubulin, protein levels of NGAL, and L-FABP after normalization to GAPDH. **(F)** Representative immunofluorometric images of NLRP3 (bar = 100 µm). **(G)** Mean fluorescence intensity of NLRP3 in kidney tissue. **(H–L)** The mRNA levels of IL-1β, IL-6, TNF-α, MCP1, and HIF-1a were determined by RT-qPCR. All data are presented as the mean ± SD (*n* = 6). ^##^ *p* < 0.01 vs. Control; ^**^ *p* < 0.01 vs. Model.

In order to confirm the impact of AS-IV on kidney damage, kidney tissue was subjected to H&E, PAS, and Masson staining. In comparison to the control group, rats in the DKD model group displayed kidney damage, which included enlarged glomeruli, noticeable growth of mesangial cells, elevated deposition of extracellular matrix (ECM), fibrosis in the interstitium, swelling of tubular epithelial cells, vacuolar degeneration, and loss of brush border (Figure 3A). Nevertheless, these anomalous alterations were mitigated in both the AS-IV treatment groups and the atorvastatin treatment group. Furthermore, a notable improvement was observed in the groups that received 40 and 80 µM AS-IV.

Mori et al. found that renal tubular cells take up fatty acids through Kidney Injury Molecule-1 (KIM-1), which leads to tubule injury accompanied by interstitial inflammation and fibrosis in DN (Mori et al., 2021). Kidney injury molecule 1 (KIM-1), liver fatty acid-binding protein (L-FABP), and neutrophil gelatinase-associated lipocalin (NGAL) are urinary biomarkers of renal tubular injury (Fufaa et al., 2015). The protein levels of KIM-1, NGAL, and L-FABP were significantly increased in DKD rats compared to the control group, as shown by Western blot analysis (Figures 3B–E). Additionally, the protein levels of KIM-1, L-FABP, and NGAL were decreased in the groups that received 40 and 80 µM AS-IV treatment. Taken together, these results showed that AS-IV could alleviate renal tubular injury in DKD.

Figures 3F, G demonstrates a notable decrease in NLRP3 expression in both the AS-IV treatment groups and the atorvastatin treatment group, as indicated by immunofluorescence staining for NLRP3. Furthermore, this reduction was particularly pronounced in the 40 µM group.

PCR analysis was conducted to determine the mRNA levels of IL-1β, IL-6, TNF-α, and MCP1 in order to explore the protective mechanism of AS-IV against DKD. According to Figures 3H–K, the mRNA levels of IL-1β, IL-6, TNF-α, and MCP1 were notably increased in DKD rats compared to the control group (*p* < 0.01). Following a 12-week treatment with AS-IV, a significant reduction in the mRNA levels of these inflammatory indicators was noted in the kidney tissue (*p* < 0.01).

Furthermore, our study found that HIF-1a expression was significantly elevated in rats with DKD (Figure 3L), while the HIF-1a mRNA level was significantly decreased after treatment with AS-IV or atorvastatin (*p* < 0.01). Growing evidence indicated that the effect of hypoxia and hypoxia-inducible factor-1 (HIF-1α) on renal tubules plays a critical role in the progression of DKD (Sun et al., 2012; Jiang et al., 2020; Feng et al., 2021). Additionally, hypoxia and HIF-1α promote podocyte injury and contribute to proteinuria (Nakuluri et al., 2019a; Nakuluri et al., 2019b). This illustrated that the mechanism by which AS-IV alleviates renal injury in DKD may be related to improving renal hypoxia.

### 3.4 AS-IV inhibits NLRP3 inflammasome activation and the upregulation of CD36 in DKD rats

**FIGURE 4:**
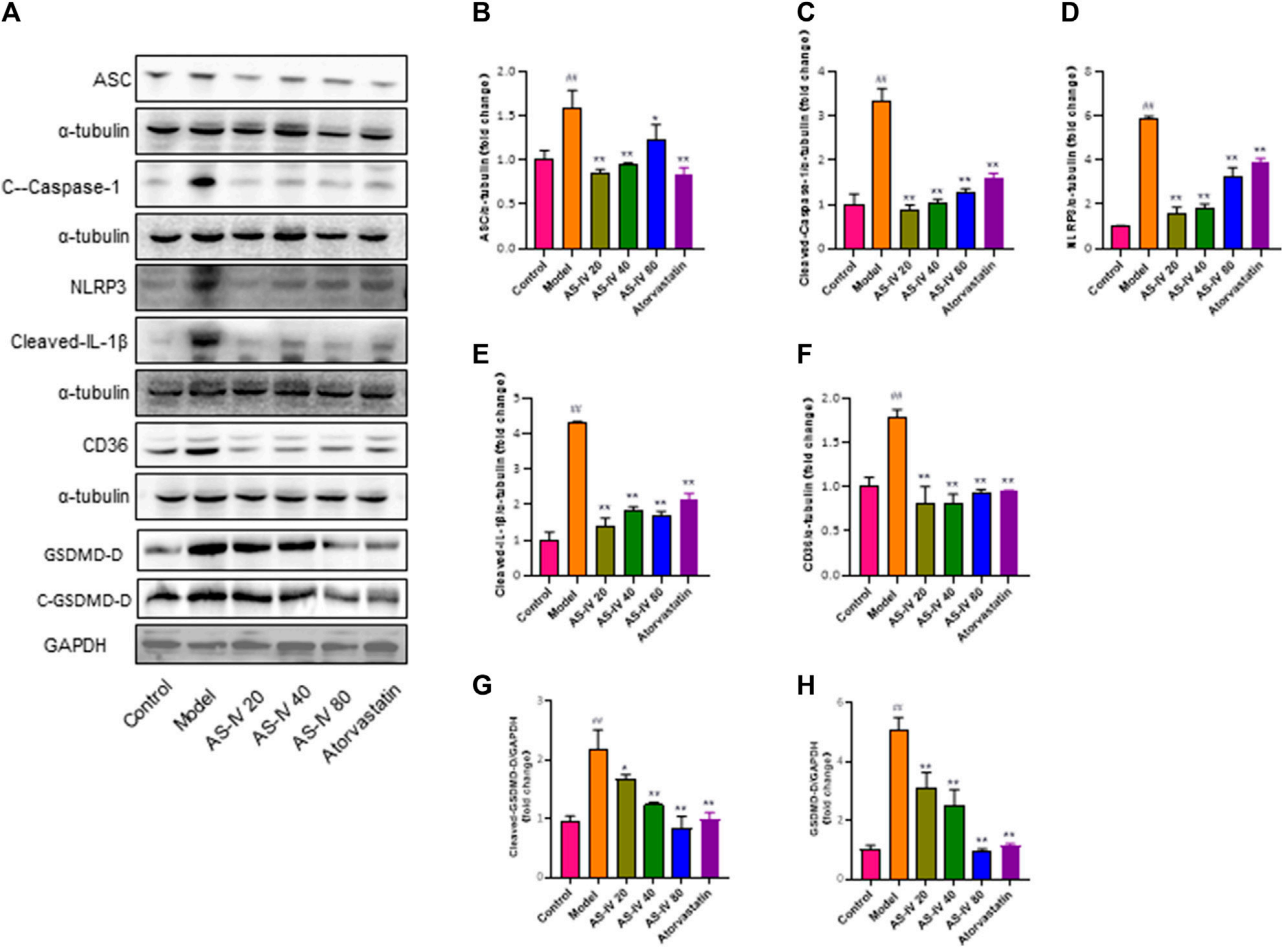
AS-IV inhibits CD36 production and NLRP3 inflammasome activation in DKD rats. **(A)** Western blot analysis of ASC, Cleaved-Caspase-1, NLRP3, Cleaved-IL-1β, CD36, GSDMD-D, and Cleaved-GSDMD-D. **(B–H)** Protein levels of ASC, Cleaved-Caspase-1, NLRP3, Cleaved-IL-1β, and CD36 after normalization to α-tubulin, protein levels of GSDMD-D, and Cleaved-GSDMD-D after normalization to GAPDH. All data are presented as the mean ± SD (*n* = 6). ^##^ *p* < 0.01 vs. Control; ^*^ *p* < 0.05 and ^**^ *p* < 0.01 vs. Model.

The protein levels of CD36, NLRP3, ASC, cleaved-Caspase-1, cleaved-IL-1β, cleaved-GSDMD-D, and GSDMD-D were analyzed to further elucidate the relationship between NLRP3 inflammasome activation and CD36 upregulation through western blot analysis using kidney tissue samples (Figures 4A–H). Our findings revealed that DKD rats exhibited significantly higher protein levels of CD36, NLRP3, ASC, cleaved-caspase-1, cleaved-IL-1β, cleaved-GSDMD-D, and GSDMD-D compared to the control group. However, the expression of these proteins was significantly decreased after treatment with AS-IV or atorvastatin (*p* < 0.01).

### 3.5 AS-IV prevents the reduction in cell viability, activation of the NLRP3 inflammasome, secretion of IL-1β, and activation of caspase-1 in PA-induced HK-2 cells

**FIGURE 5:**
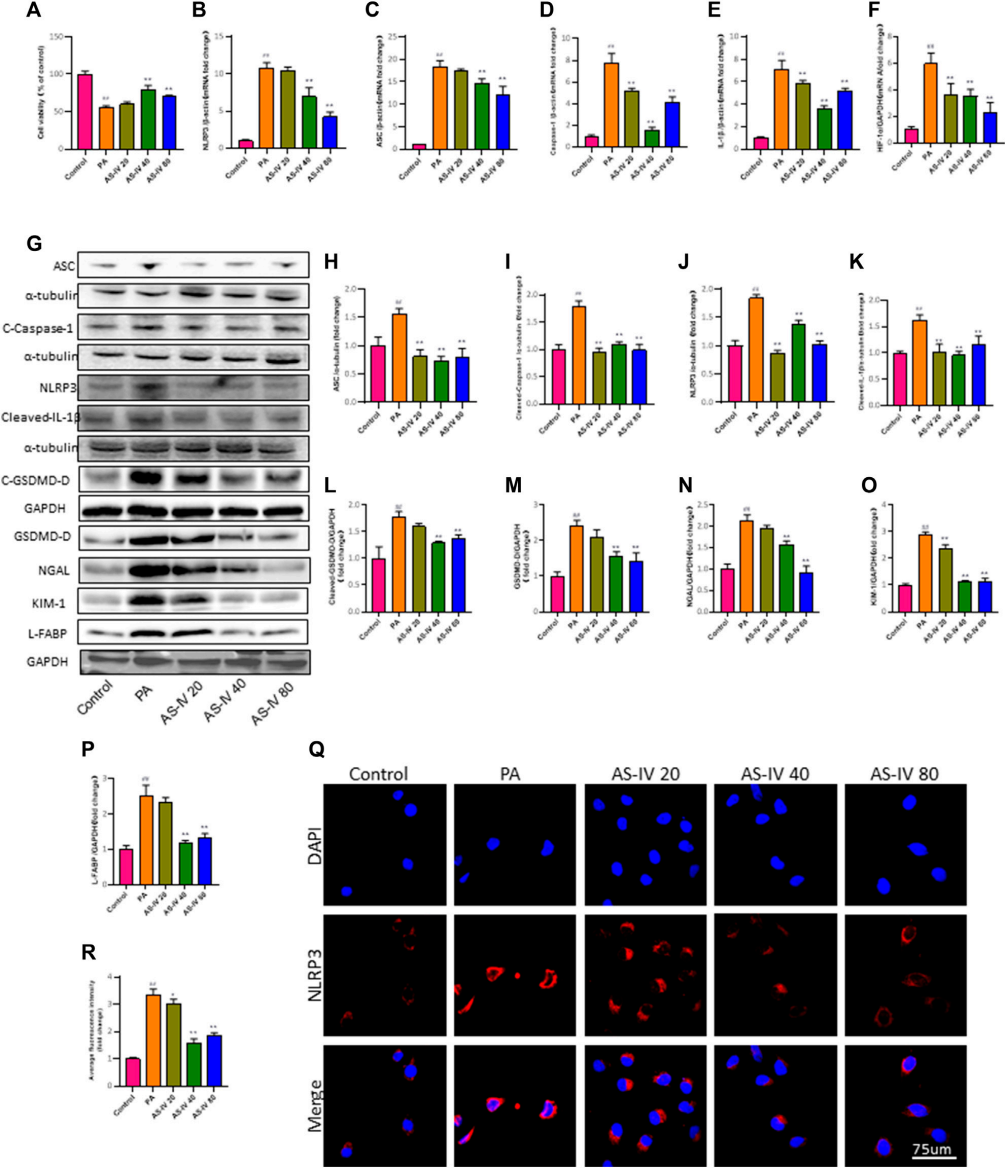
AS-IV inhibits the PA-induced decrease in cell viability and NLRP3 inflammasome activation in HK-2 cells. **(A)** HK-2 cells were cultured with different concentrations of AS-IV for 24 h and cell viability was assessed using the CCK-8 assay. **(B–F)** The mRNA expression levels of NLRP3, ASC, Caspase-1, IL-1β, and HIF-1a were determined by RT-qPCR. **(G)** Western blot analysis of ASC, Cleaved-Caspase-1, NLRP3, Cleaved-IL-1β, GSDMD-D, Cleaved-GSDMD-D, NGAL, KIM-1, and L-FABP. **(H–P)** The protein levels of ASC, Cleaved-Caspase-1, NLRP3, and Cleaved-IL-1β after normalization to α-tubulin, the protein levels of GSDMD-D, Cleaved-GSDMD-D, NGAL, KIM-1 and L-FABP after normalization to GAPDH. **(Q,R)** Immunofluorescence staining of NLRP3 in each group. Data are expressed as the mean ± SD. ^##^ *p* < 0.01 vs. Control; ^*^ *p* < 0.05 and ^**^ *p* < 0.01 vs. PA.

The CCK-8 assay was used to assess the effect of AS-IV on the cytotoxicity of PA in HK-2 cells. Following a 24-h exposure to PA, there was a notable decline in cell viability, which was restored by AS-IV at concentrations of 40 and 80 µM (*p* < 0.01). Nevertheless, treatment with 20 µM AS-IV did not have a noteworthy impact on cell viability (*p* > 0.05). These findings suggest that PA can induce cell death in HK-2 cells and this effect can be alleviated with AS-IV at proper concentrations (Figure 5A).

Furthermore, PCR and western blot were utilized to assess the mRNA and protein levels of NLRP3, ASC, Caspase-1, and IL-1β in order to investigate the impact of AS-IV treatment on PA-induced HK-2 cells. As depicted in Figures 5B–F, the mRNA levels of NLRP3, ASC, Caspase-1, IL-1β, and HIF-1a in PA-induced HK-2 cells were significantly higher compared to the control group (*p* < 0.01). However, treatment with AS-IV at concentrations of 40 and 80 µM resulted in a significant decrease in these levels (*p* < 0.01). Additionally, the protein levels of NLRP3, ASC, cleaved-Caspase-1, cleaved-IL-1β, cleaved-GSDMD-D, GSDMD, KIM-1, NGAL, and L-FABP were evaluated. As shown in Figures 5G–P, the protein levels of these factors were elevated compared to the control group. However, they were notably diminished after AS-IV treatment (*p* < 0.01).

After treatment with AS-IV, the immunofluorescence staining for NLRP3 in HK-2 cells was significantly improved, as shown in Figures 5Q, R. The results indicate that the activation of the NLRP3 inflammasome is linked to cell demise caused by PA, whereas AS-IV has the ability to block this.

### 3.6 AS-IV inhibits CD36 expression, intracellular lipid accumulation, and ROS production in HK-2 cells

**FIGURE 6:**
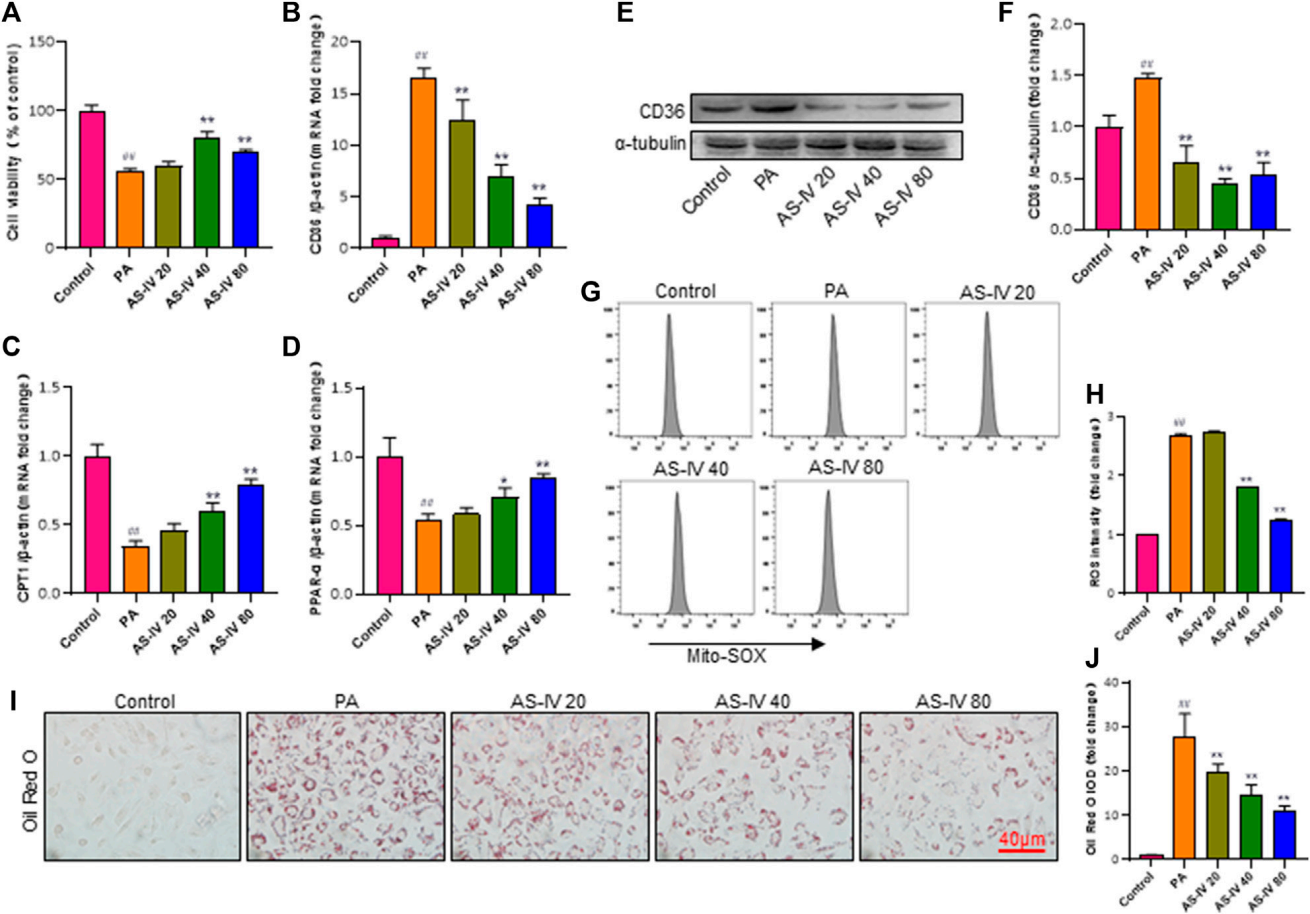
AS-IV inhibits CD36 expression, lipid accumulation, and lipid ROS production in PA-induced HK-2 cells. **(B–D)** The mRNA expression levels of CD36, CPT1, and PPAP-α were determined by RT-qPCR. **(E,F)** The protein levels of CD36 were determined by western blot and normalized to α-tubulin. **(G,H)** Lipid ROS production was detected by flow cytometric analysis. **(I)** Lipid accumulation in each group was assessed by Oil Red O (ORO) staining under light microscopy (bar = 40 μm). **(J)** Densitometric analysis of ORO staining. Data are expressed as the mean ± SD. ^##^ *p* < 0.01 vs. Control; ^*^ *p* < 0.05 and ^**^ *p* < 0.01 vs. PA.

Previous research has demonstrated that CD36 potentially assumes a pivotal function in metabolic kidney disorders, while the accumulation of lipids facilitated by CD36 may be linked to activation of the NLRP3 inflammasome. In order to further examine the mechanism by which AS-IV suppresses the activation of the NLRP3 inflammasome, we measured the levels of CD36 through quantitative RT-PCR and western blot analysis. As illustrated in Figures 6B, E, F, the levels of CD36 were significantly higher in the PA groups compared to the control group (*p* < 0.01), but they were significantly decreased after AS-IV treatment. CD36 is a crucial molecule involved in lipid transport. To confirm the association between CD36 and lipid metabolism, we investigated the expression levels of CPT1 and PPAR-α, along with ROS production. Additionally, intracellular neutral lipid accumulation was assessed using Oil Red O staining. Exposure to PA resulted in increased levels of lipid ROS and intracellular lipid accumulation, simultaneously reducing the expression of CPT1 and PPAR-α (Figures 6C, D, G, H, *p* < 0.01). However, these effects were significantly reversed following AS-IV treatment. The results suggest that PA-stimulated HK-2 cells exhibit elevated levels of both lipid ROS and CD36; however, AS-IV effectively attenuated CD36 expression and inhibited lipid ROS production. Recent studies (Sheedy et al., 2013; Dai et al., 2017) have demonstrated that CD36 triggers the NLRP3 inflammasome in atherosclerosis, while inhibition of CD36 suppresses NLRP3 inflammasome activation (Kang et al., 2016). Taken together, we hypothesize that AS-IV might alleviate the activation of the NLRP3 inflammasome by suppressing the expression of CD36 in PA-induced HK-2 cells.

### 3.7 AS-IV protects renal function through inhibiting CD36 expression, lipid peroxidation, and ROS production

**FIGURE 7:**
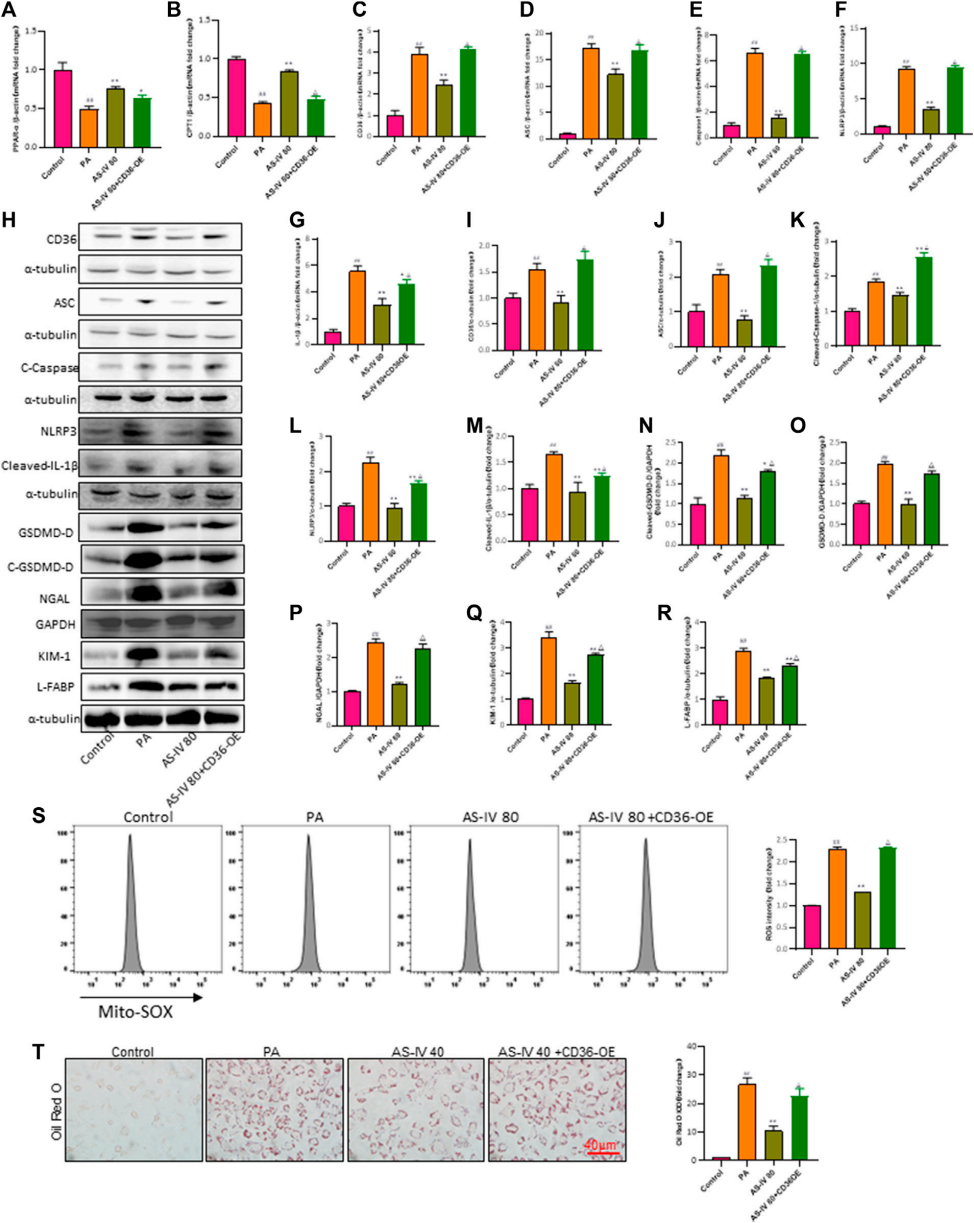
Overexpression of CD36 promotes PA-induced lipid accumulation, lipid ROS production, and activation of the NLRP3 inflammatory body and reverses the protective effect of AS-IV on the expression of lipid peroxidation-related proteins and the NLRP3 inflammatory body in PA-induced HK-2 cells. **(A–G)** The mRNA expression levels of PPAP-α, CPT1, CD36, ASC, Caspase-1, NLRP3, and IL-1β were determined by RT-qPCR. **(H–R)** Western blot analysis was conducted to determine the expression of CD36, ASC, Cleaved-Caspase-1, NLRP3, Cleaved-IL-1β, GSDMD-D, Cleaved-GSDMD-D, NGAL, KIM-1 and L-FABP. **(S)** Lipid ROS production was detected by flow cytometric analysis. **(T)** Lipid accumulation was assessed by ORO staining under light microscopy (bar = 40 μm). Data are expressed as the mean ± SD. ^##^ *p* < 0.01 vs. Control; ^*^ *p* < 0.05 and ^**^ *p* < 0.01 vs. PA; ^△^ *p* < 0.05 vs. PA + AS-IV 80.

To further confirm the protective impact of AS-IV through its inhibition of CD36, we conducted a rescue experiment by introducing a CD36 lentiviral vectors (adenovirus) to enhance CD36 expression in PA-induced HK-2 cells. CD36 overexpression diminished the suppressive impact of AS-IV on intracellular lipid buildup and ROS (Figures 7S, T, *p* < 0.01). Additionally, compared with other groups, the expression levels of CD36, NLRP3, ACS, Caspase-1, and IL-1β were significantly elevated in the CD36 overexpression groups. Notably, AS-IV treatment effectively suppressed the PA-induced upregulation of CD36, NLRP3, ASC, Caspase-1, and IL-1β; however, these inhibitory effects were reversed upon CD36 overexpression (Figures 7C–R, *p* < 0.01). Collectively, these findings indicate that AS-IV mitigates PA-induced HK-2 cell injury through inhibiting CD36 expression.

## 4 Discussion

Diabetes, a prevalent and significant chronic illness, is a crucial global public health concern. According to the 10th edition of International Diabetes Federation (IDF) (Sun et al., 2022), the number of adults aged 20–79 suffering from diabetes in 2021 was approximately 536.6 million, and it is anticipated to escalate to 783.2 million by 2045. With the increasing number of individuals with diabetes, the prevalence of DKD, a widely acknowledged microvascular complication linked to diabetes, is also rising. Currently, the therapeutic approaches for DKD are mainly focused on glycemic control and blood pressure management; however, there are still no effective therapeutic measures against the progression of DKD, indicating potential involvement of alternative mechanisms (Falkevall et al., 2017). Therefore, it is imperative to explore more effective methods for reversing DKD.

An increasing body of evidence indicates that the release of multiple inflammatory factors is a profoundly pathological phenomenon of DN (Perlman et al., 2015). Both glomerulosclerosis and renal tubular fibrosis of DKD are associated with renal inflammation (Mori et al., 2021). Studies have revealed that tubular epithelial cells play a critical role in driving interstitial inflammation, in addition to the local inflammatory cascade response in DKD (Liu et al., 2018). Also, it has been confirmed that renal tubulointerstitial inflammation, especially proximal tubular epithelial cell inflammation, plays a significant role in the pathogenesis of DN (Tang and Lai, 2012). Therefore, inhibition of renal tubular inflammation might be a novel therapeutic strategy to slow down the progression of DKD. However, pharmacological interventions aimed at preserving renal tubular cells have not yet achieved clinical significance. Therefore, traditional Chinese medicine herbs are regarded as a cost-effective alternative approach.

AS-IV, a key bioactive pharmacological constituent of Astragalus membranaceus (Fisch.) (Hao et al., 2018), has been documented for its efficacy in treating diabetes and associated complications, including DKD, cardiovascular diseases, and diabetic retinopathy (Ju et al., 2019; Zhang et al., 2019). Several preclinical investigations have suggested that AS-IV has the capacity to inhibit the inflammatory reaction in diabetic nephropathy. Gui *et al.* (Gui et al., 2013) found that AS-IV alleviated renal injury and fibrosis by suppressing the expression of inflammatory genes mediated by NF-κB in streptozotocin-induced diabetic rats. Xing (Xing et al., 2021) suggested that AS-IV could potentially reduce podocyte apoptosis by inhibiting oxidative stress through activation of the PPARγ–Klotho–FoxO1 signaling pathway. Nevertheless, there is currently a lack of comprehensive pharmacological evaluations regarding the therapeutic effects of AS-IV on DKD.

In this study, the levels of FBG, TC, TG, and LDL-C all exhibited an increase in DKD rats compared to the control group. In addition, significant elevations in urinary albumin, ACR, BUN, and Scr indicated renal injury in DKD rats. Renal damage was confirmed by H&E, PAS, and Masson staining. These stains revealed glomerular hypertrophy, obvious mesangial proliferation, interstitium fibrosis, tubular epithelial cell swelling, and vacuolar degeneration in the kidneys of DKD rats. Moreover, renal tubular injury was additionally confirmed through the protein levels of KIM-1, L-FABP, and NGAL in the kidney tissue of DKD rats. In brief, the DKD model induced by streptozotocin and HFD was proved to be stable. Our results also demonstrated that AS-IV had a beneficial effect on improving lipid metabolism disorder and renal function in DKD rats. The buildup of lipids is a frequent occurrence in individuals diagnosed with DKD (Herman-Edelstein et al., 2014). It has been discovered that tubular lipotoxicity causes a series of renal injuries, including endoplasmic reticulum (ER) stress, oxidative stress (OS), apoptosis of tubular epithelial cells, tubulointerstitial fibrosis (TIF), mitochondrial dysfunction, and inflammation (Schelling, 2016; Jao et al., 2019; Ge et al., 2020). Several studies have demonstrated that dyslipidemia plays a substantial part in the development of DKD, and it is believed that tubular injury caused by lipotoxicity is a critical occurrence in DKD (Wang et al., 2021). In the FIELD study, we also observed that administration of fenofibrate could decrease albuminuria and reduce renal dysfunction in individuals diagnosed with type 2 diabetes (Davis et al., 2011). Nevertheless, the impact of statins (antihyperlipidemic drugs) on the advancement of diabetic nephropathy remains controversial (Sandhu et al., 2006; Colhoun et al., 2009).

Our previous study demonstrated that AS-IV can reduce the levels of TG and TC, downregulate CD36, and attenuate myocardial dysfunction in diabetic cardiomyopathy rats (Li et al., 2023). In this study, we also observed that AS-IV modulates lipid metabolism and protects kidney function. CD36, a multifunctional receptor, is mainly found in renal tubular epithelial cells and podocytes in the kidney (Yang et al., 2017). It plays a role in lipid metabolism and acts as a connection between cholesterol metabolism and inflammatory activation (Xu et al., 2018). Sheedy *et al.* (Sheedy et al., 2013) discovered that the uptake of oxidized LDL (oxLDL) through CD36 leads to the formation of crystals inside cells and triggers the activation of the NLRP3 inflammasome. This process initiates an early pathogenic pathway that connects the buildup of cholesterol to the chronic inflammatory process of atherosclerosis. Zhao *et al.* confirmed that the accumulation of lipids through CD36 and activation of the NLRP3 inflammasome could potentially contribute to the pathogenesis of ORG podocyte injury (Zhao et al., 2019). Liu and others showed that activation of the NLRP3 inflammasome and secretion of IL-1β were primarily caused by CD36-mediated production of ROS in response to oxLDL stimulation (Liu et al., 2014). Yang *et al.* reported that CD36 could potentially facilitate the cell death of podocytes by activating the NLRP3 inflammasome in primary nephrotic syndrome (Yang et al., 2018). Furthermore, recent evidence indicated that CD36-mediated NLRP3 inflammasome activation hinders mitochondrial FAO and promotes mtROS generation within renal tubular epithelial cells in DKD (Hou et al., 2021). Moreover, recent research has shown that GSDMD-mediated pyroptosis plays a significant role in the occurrence and development of DN (Zuo et al., 2021), and AS-IV could alleviate MI-induced myocardial fibrosis and cardiac remodeling by suppressing the ROS/Caspase-1/GSDMD signaling pathway (Zhang et al., 2022). In our study, we observed an increase in CD36 expression, accumulation of lipids, activation of the NLRP3 inflammasome, secretion of IL-1β, and the expression of GSDMD in PA-induced HK-2 cells and renal tissue of DKD rats. AS-IV suppressed CD36 expression, lipid accumulation, NLRP3 inflammasome activation, IL-1β secretion, and the expression of GSDMD. Additionally, tubular injury was alleviated. These findings indicate that AS-IV may have the ability to mitigate tubular damage, attenuate lipid accumulation, reduce the production of ROS, suppress the activation of the NLRP3 inflammatory body and the expression of GSDMD by downregulating CD36 expression in both PA-induced HK-2 cells and DKD rats.

In conclusion, this study showed that AS-IV effectively mitigates glucolipid metabolism disorders, alleviates renal tubular injury, and protects the kidney in DKD rats. Furthermore, AS-IV alleviated renal tubule interstitial inflammation by inhibiting CD36-mediated NLRP3 inflammasome activation in PA-induced HK-2 cells. The results suggest that AS-IV has significant potential as a viable treatment choice for DKD. Certainly, the molecular mechanism by which AS-IV alleviates DKD may be more complex than described in our study, and our data does not exclude the potential involvement of any other mechanisms underlying the beneficial effects of AS-IV such as protective effects on podocytes and effects unrelated to renal tubular function. However, the in-vitro results presented in the study do help define that AS-IV reduced inflammatory reaction in PA-induced HK-2 cells, and CD36-ROS-NLRP3 signaling is probably a new mechanism central to renal tubular protection in DKD. Taken together, our findings provide novel mechanistic insights into the protective role of AS-IV and may contribute to the development of new therapies for DKD.

## Data Availability

The datasets presented in this study can be found in online repositories. The names of the repository/repositories and accession number(s) can be found in the article/Supplementary Material.

## References

[B1] Chan G. Tang S. C. (2013). Current practices in the management of diabetic nephropathy. J. R. Coll. Physicians Edinb 43, 330–332. quiz 333. 10.4997/jrcpe.2013.413 24350318

[B2] Chen Q. Su Y. Ju Y. Ma K. Li W. Li W. (2018). Astragalosides IV protected the renal tubular epithelial cells from free fatty acids-induced injury by reducing oxidative stress and apoptosis. Biomed. Pharmacother. 108, 679–686. 10.1016/j.biopha.2018.09.049 30245468

[B3] Colhoun H. M. Betteridge D. J. Durrington P. N. Hitman G. A. Neil H. A. Livingstone S. J. (2009). Effects of atorvastatin on kidney outcomes and cardiovascular disease in patients with diabetes: an analysis from the Collaborative Atorvastatin Diabetes Study (CARDS). Am. J. Kidney Dis. 54, 810–819. 10.1053/j.ajkd.2009.03.022 19540640

[B4] Dai Y. Cao Y. Zhang Z. Vallurupalli S. Mehta J. L. (2017). Xanthine oxidase induces foam cell formation through LOX-1 and NLRP3 activation. Cardiovasc Drugs Ther. 31, 19–27. 10.1007/s10557-016-6706-x 28084571

[B5] Davis T. M. Ting R. Best J. D. Donoghoe M. W. Drury P. L. Sullivan D. R. (2011). Effects of fenofibrate on renal function in patients with type 2 diabetes mellitus: the Fenofibrate Intervention and Event Lowering in Diabetes (FIELD) Study. Diabetologia 54, 280–290. 10.1007/s00125-010-1951-1 21052978

[B6] Duewell P. Kono H. Rayner K. J. Sirois C. M. Vladimer G. Bauernfeind F. G. (2010). NLRP3 inflammasomes are required for atherogenesis and activated by cholesterol crystals. Nature 464, 1357–1361. 10.1038/nature08938 20428172 PMC2946640

[B7] Falkevall A. Mehlem A. Palombo I. Heller Sahlgren B. Ebarasi L. He L. (2017). Reducing VEGF-B signaling ameliorates renal lipotoxicity and protects against diabetic kidney disease. Cell Metab. 25, 713–726. 10.1016/j.cmet.2017.01.004 28190774

[B8] Feng X. Wang S. Sun Z. Dong H. Yu H. Huang M. (2021). Ferroptosis enhanced diabetic renal tubular injury via HIF-1α/HO-1 pathway in db/db mice. Front. Endocrinol. (Lausanne) 12, 626390. 10.3389/fendo.2021.626390 33679620 PMC7930496

[B9] Fufaa G. D. Weil E. J. Nelson R. G. Hanson R. L. Bonventre J. V. Sabbisetti V. (2015). Association of urinary KIM-1, L-FABP, NAG and NGAL with incident end-stage renal disease and mortality in American Indians with type 2 diabetes mellitus. Diabetologia 58, 188–198. 10.1007/s00125-014-3389-3 25316431 PMC4258130

[B10] Ge M. Fontanesi F. Merscher S. Fornoni A. (2020). The vicious cycle of renal lipotoxicity and mitochondrial dysfunction. Front. Physiol. 11, 732. 10.3389/fphys.2020.00732 32733268 PMC7358947

[B11] Gui D. Huang J. Guo Y. Chen J. Chen Y. Xiao W. (2013). Astragaloside IV ameliorates renal injury in streptozotocin-induced diabetic rats through inhibiting NF-κB-mediated inflammatory genes expression. Cytokine 61, 970–977. 10.1016/j.cyto.2013.01.008 23434274

[B12] Hao M. Liu Y. Chen P. Jiang H. Kuang H. Y. (2018). Astragaloside IV protects RGC-5 cells against oxidative stress. Neural Regen. Res. 13, 1081–1086. 10.4103/1673-5374.233452 29926836 PMC6022471

[B13] Herman-Edelstein M. Scherzer P. Tobar A. Levi M. Gafter U. (2014). Altered renal lipid metabolism and renal lipid accumulation in human diabetic nephropathy. J. Lipid Res. 55, 561–572. 10.1194/jlr.P040501 24371263 PMC3934740

[B14] Hou Y. Wang Q. Han B. Chen Y. Qiao X. Wang L. (2021). CD36 promotes NLRP3 inflammasome activation via the mtROS pathway in renal tubular epithelial cells of diabetic kidneys. Cell Death Dis. 12, 523. 10.1038/s41419-021-03813-6 34021126 PMC8140121

[B15] Hua W. Huang H. Z. Tan L. T. Wan J. M. Gui H. B. Zhao L. (2015). CD36 mediated fatty acid-induced podocyte apoptosis via oxidative stress. PLoS One 10, e0127507. 10.1371/journal.pone.0127507 26000608 PMC4441449

[B16] Jao T. M. Nangaku M. Wu C. H. Sugahara M. Saito H. Maekawa H. (2019). ATF6α downregulation of PPARα promotes lipotoxicity-induced tubulointerstitial fibrosis. Kidney Int. 95, 577–589. 10.1016/j.kint.2018.09.023 30639234

[B17] Jiang N. Zhao H. Han Y. Li L. Xiong S. Zeng L. (2020). HIF-1α ameliorates tubular injury in diabetic nephropathy via HO-1-mediated control of mitochondrial dynamics. Cell Prolif. 53, e12909. 10.1111/cpr.12909 32975326 PMC7653251

[B18] Ju Y. Su Y. Chen Q. Ma K. Ji T. Wang Z. (2019). Protective effects of Astragaloside IV on endoplasmic reticulum stress-induced renal tubular epithelial cells apoptosis in type 2 diabetic nephropathy rats. Biomed. Pharmacother. 109, 84–92. 10.1016/j.biopha.2018.10.041 30396095

[B19] Kang L. L. Zhang D. M. Ma C. H. Zhang J. H. Jia K. K. Liu J. H. (2016). Cinnamaldehyde and allopurinol reduce fructose-induced cardiac inflammation and fibrosis by attenuating CD36-mediated TLR4/6-IRAK4/1 signaling to suppress NLRP3 inflammasome activation. Sci. Rep. 6, 27460. 10.1038/srep27460 27270216 PMC4897702

[B20] Kanwar Y. S. Sun L. Xie P. Liu F. Y. Chen S. (2011). A glimpse of various pathogenetic mechanisms of diabetic nephropathy. Annu. Rev. Pathol. 6, 395–423. 10.1146/annurev.pathol.4.110807.092150 21261520 PMC3700379

[B21] Li X. Li Z. Dong X. Wu Y. Li B. Kuang B. (2023). Astragaloside IV attenuates myocardial dysfunction in diabetic cardiomyopathy rats through downregulation of CD36-mediated ferroptosis. Phytother. Res. 37, 3042–3056. 10.1002/ptr.7798 36882189

[B22] Liu B. C. Tang T. T. Lv L. L. Lan H. Y. (2018). Renal tubule injury: a driving force toward chronic kidney disease. Kidney Int. 93, 568–579. 10.1016/j.kint.2017.09.033 29361307

[B23] Liu W. Yin Y. Zhou Z. He M. Dai Y. (2014). OxLDL-induced IL-1 beta secretion promoting foam cells formation was mainly via CD36 mediated ROS production leading to NLRP3 inflammasome activation. Inflamm. Res. 63, 33–43. 10.1007/s00011-013-0667-3 24121974

[B24] Mori Y. Ajay A. K. Chang J. H. Mou S. Zhao H. Kishi S. (2021). KIM-1 mediates fatty acid uptake by renal tubular cells to promote progressive diabetic kidney disease. Cell Metab. 33, 1042–1061.e7. 10.1016/j.cmet.2021.04.004 33951465 PMC8132466

[B25] Nakuluri K. Mukhi D. Mungamuri S. K. Pasupulati A. K. (2019a). Stabilization of hypoxia-inducible factor 1α by cobalt chloride impairs podocyte morphology and slit-diaphragm function. J. Cell Biochem. 120, 7667–7678. 10.1002/jcb.28041 30387200

[B26] Nakuluri K. Mukhi D. Nishad R. Saleem M. A. Mungamuri S. K. Menon R. K. (2019b). Hypoxia induces ZEB2 in podocytes: implications in the pathogenesis of proteinuria. J. Cell Physiol. 234, 6503–6518. 10.1002/jcp.27387 30238984

[B27] Navarro-González J. F. Mora-Fernández C. Muros De Fuentes M. García-Pérez J. (2011). Inflammatory molecules and pathways in the pathogenesis of diabetic nephropathy. Nat. Rev. Nephrol. 7, 327–340. 10.1038/nrneph.2011.51 21537349

[B28] Perlman A. S. Chevalier J. M. Wilkinson P. Liu H. Parker T. Levine D. M. (2015). Serum inflammatory and immune mediators are elevated in early stage diabetic nephropathy. Ann. Clin. Lab. Sci. 45, 256–263. 26116588

[B29] Qiu Y. Y. Tang L. Q. (2016). Roles of the NLRP3 inflammasome in the pathogenesis of diabetic nephropathy. Pharmacol. Res. 114, 251–264. 10.1016/j.phrs.2016.11.004 27826011

[B30] Sandhu S. Wiebe N. Fried L. F. Tonelli M. (2006). Statins for improving renal outcomes: a meta-analysis. J. Am. Soc. Nephrol. 17, 2006–2016. 10.1681/asn.2006010012 16762986

[B31] Schelling J. R. (2016). Tubular atrophy in the pathogenesis of chronic kidney disease progression. Pediatr. Nephrol. 31, 693–706. 10.1007/s00467-015-3169-4 26208584 PMC4726480

[B32] Sheedy F. J. Grebe A. Rayner K. J. Kalantari P. Ramkhelawon B. Carpenter S. B. (2013). CD36 coordinates NLRP3 inflammasome activation by facilitating intracellular nucleation of soluble ligands into particulate ligands in sterile inflammation. Nat. Immunol. 14, 812–820. 10.1038/ni.2639 23812099 PMC3720827

[B33] Stadler K. Goldberg I. J. Susztak K. (2015). The evolving understanding of the contribution of lipid metabolism to diabetic kidney disease. Curr. Diab Rep. 15, 40. 10.1007/s11892-015-0611-8 25957525 PMC4548922

[B34] Su Y. Chen Q. Ma K. Ju Y. Ji T. Wang Z. (2019). Astragaloside IV inhibits palmitate-mediated oxidative stress and fibrosis in human glomerular mesangial cells via downregulation of CD36 expression. Pharmacol. Rep. 71, 319–329. 10.1016/j.pharep.2018.12.008 30826573

[B35] Sun H. Saeedi P. Karuranga S. Pinkepank M. Ogurtsova K. Duncan B. B. (2022). IDF Diabetes Atlas: global, regional and country-level diabetes prevalence estimates for 2021 and projections for 2045. Diabetes Res. Clin. Pract. 183, 109119. 10.1016/j.diabres.2021.109119 34879977 PMC11057359

[B36] Sun H. K. Lee Y. M. Han K. H. Kim H. S. Ahn S. H. Han S. Y. (2012). Phosphodiesterase inhibitor improves renal tubulointerstitial hypoxia of the diabetic rat kidney. Korean J. Intern Med. 27, 163–170. 10.3904/kjim.2012.27.2.163 22707888 PMC3372800

[B37] Tang S. C. Lai K. N. (2012). The pathogenic role of the renal proximal tubular cell in diabetic nephropathy. Nephrol. Dial. Transpl. 27, 3049–3056. 10.1093/ndt/gfs260 22734110

[B38] Vallon V. (2011). The proximal tubule in the pathophysiology of the diabetic kidney. Am. J. Physiol. Regul. Integr. Comp. Physiol. 300, R1009–R1022. 10.1152/ajpregu.00809.2010 21228342 PMC3094037

[B39] Wang H. Zhang S. Guo J. (2021). Lipotoxic proximal tubular injury: a primary event in diabetic kidney disease. Front. Med. (Lausanne) 8, 751529. 10.3389/fmed.2021.751529 34760900 PMC8573085

[B40] Wu M. Han W. Song S. Du Y. Liu C. Chen N. (2018). NLRP3 deficiency ameliorates renal inflammation and fibrosis in diabetic mice. Mol. Cell Endocrinol. 478, 115–125. 10.1016/j.mce.2018.08.002 30098377

[B41] Xing L. Fang J. Zhu B. Wang L. Chen J. Wang Y. (2021). Astragaloside IV protects against podocyte apoptosis by inhibiting oxidative stress via activating PPARγ-Klotho-FoxO1 axis in diabetic nephropathy. Life Sci. 269, 119068. 10.1016/j.lfs.2021.119068 33476631

[B42] Xu C. Zhang C. Ji J. Wang C. Yang J. Geng B. (2018). CD36 deficiency attenuates immune-mediated hepatitis in mice by modulating the proapoptotic effects of CXC chemokine ligand 10. Hepatology 67, 1943–1955. 10.1002/hep.29716 29220536

[B43] Yang X. Okamura D. M. Lu X. Chen Y. Moorhead J. Varghese Z. (2017). CD36 in chronic kidney disease: novel insights and therapeutic opportunities. Nat. Rev. Nephrol. 13, 769–781. 10.1038/nrneph.2017.126 28919632

[B44] Yang X. Wu Y. Li Q. Zhang G. Wang M. Yang H. (2018). CD36 promotes podocyte apoptosis by activating the pyrin domain-containing-3 (NLRP3) inflammasome in primary nephrotic syndrome. Med. Sci. Monit. 24, 6832–6839. 10.12659/msm.909810 30258045 PMC6178869

[B45] Yokoi H. Yanagita M. (2016). Targeting the fatty acid transport protein CD36, a class B scavenger receptor, in the treatment of renal disease. Kidney Int. 89, 740–742. 10.1016/j.kint.2016.01.009 26994570

[B46] Zhang X. Qu H. Yang T. Liu Q. Zhou H. (2022). Astragaloside IV attenuate MI-induced myocardial fibrosis and cardiac remodeling by inhibiting ROS/caspase-1/GSDMD signaling pathway. Cell Cycle 21, 2309–2322. 10.1080/15384101.2022.2093598 35770948 PMC9586672

[B47] Zhang Z. Wang J. Zhu Y. Zhang H. Wang H. (2019). Astragaloside IV alleviates myocardial damage induced by type 2 diabetes via improving energy metabolism. Mol. Med. Rep. 20, 4612–4622. 10.3892/mmr.2019.10716 31702040 PMC6797977

[B48] Zhao J. Rui H. L. Yang M. Sun L. J. Dong H. R. Cheng H. (2019). CD36-Mediated lipid accumulation and activation of NLRP3 inflammasome lead to podocyte injury in obesity-related glomerulopathy. Mediat. Inflamm. 2019, 3172647. 10.1155/2019/3172647 PMC6487104 31097920

[B49] Zhou X. Sun X. Gong X. Yang Y. Chen C. Shan G. (2017). Astragaloside IV from Astragalus membranaceus ameliorates renal interstitial fibrosis by inhibiting inflammation via TLR4/NF-кB *in vivo* and *in vitro* . Int. Immunopharmacol. 42, 18–24. 10.1016/j.intimp.2016.11.006 27855303

[B50] Zuo Y. Chen L. Gu H. He X. Ye Z. Wang Z. (2021). GSDMD-mediated pyroptosis: a critical mechanism of diabetic nephropathy. Expert Rev. Mol. Med. 23, e23. 10.1017/erm.2021.27 34955116 PMC8724266

