# Supplementary material for: Astragaloside IV attenuates renal tubule injury in DKD rats via suppression of CD36-mediated NLRP3 inflammasome activation
Source: Front Pharmacol. 2024 Mar 20;15:1285797. doi: 10.3389/fphar.2024.1285797 (PMC10987761; doi:10.3389/fphar.2024.1285797)

Fig3- KIM-1

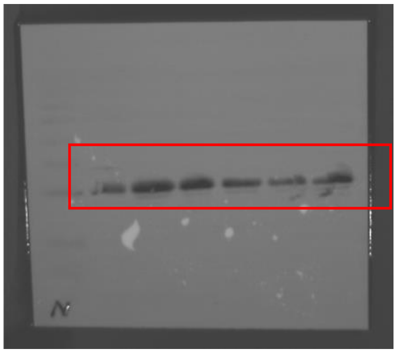

Fig3- a-tubulin (KIM-1)

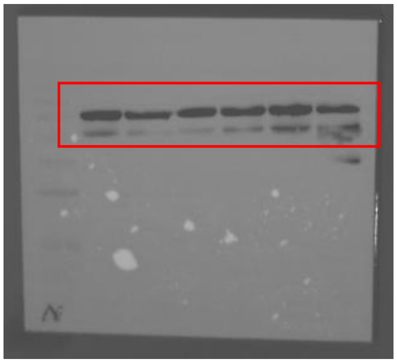

Fig3- NGAL

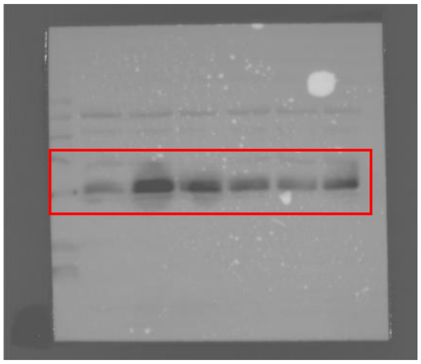

Fig3- L-FABP

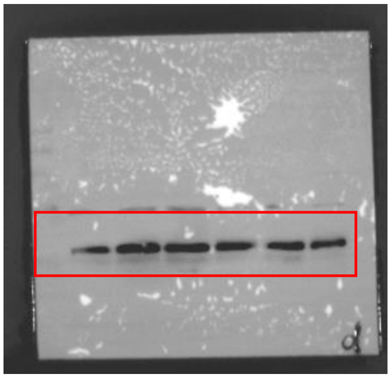

Fig3- GAPDH

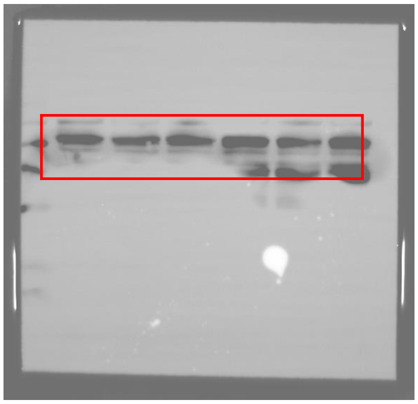

Fig4- C-GSDMD-D

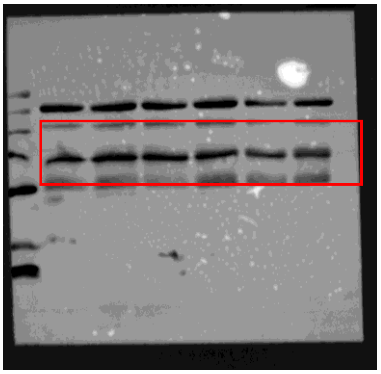

Fig4- GSDMD-D

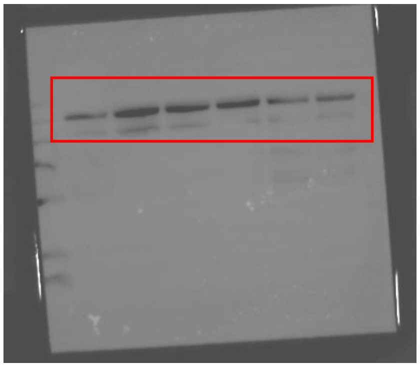

Fig4- GAPDH

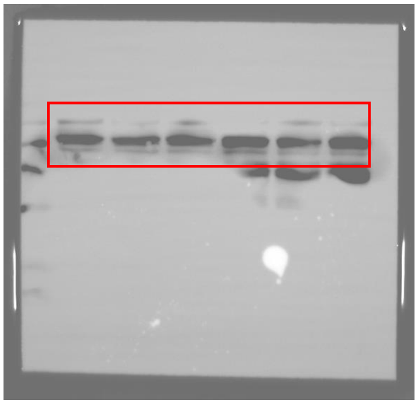

Fig4- cleaved-IL-1B

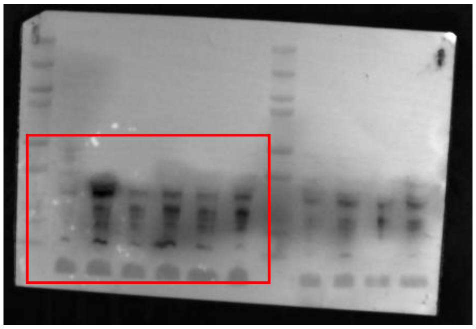

Fig4-NLRP3

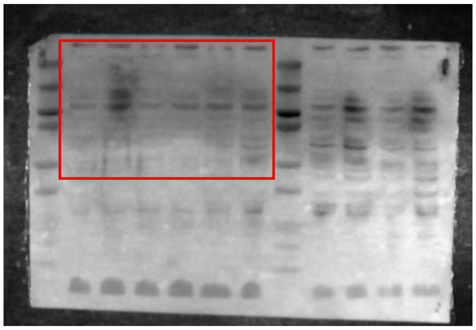

Fig4-a-tubulin (IL-1B/NLRP3

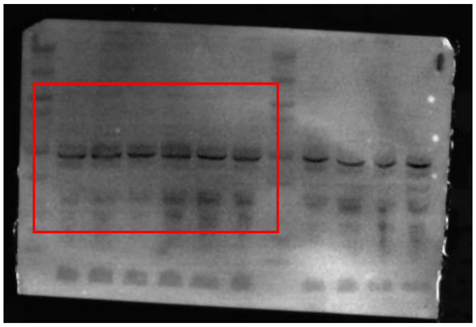

Fig4- ASC

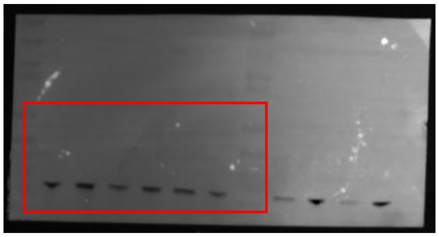

Fig4- a-tubulin (ASC)

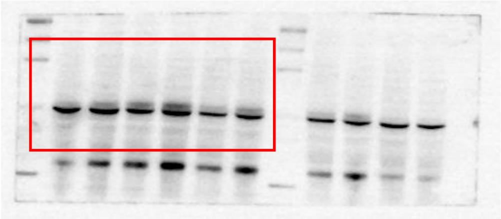

Fig4- cleaved-Caspsae1

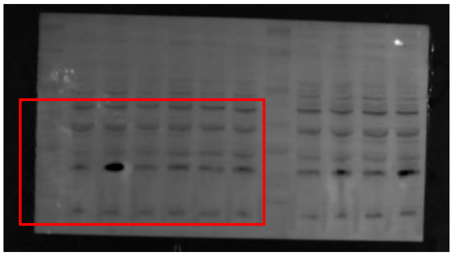

Fig4- a-tubulin (cleaved-Caspase1)

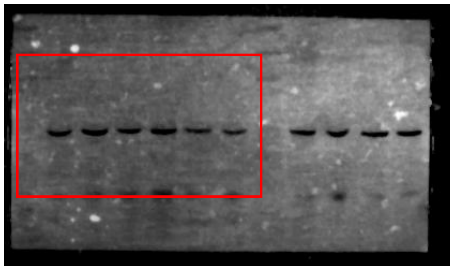

Fig4- CD36

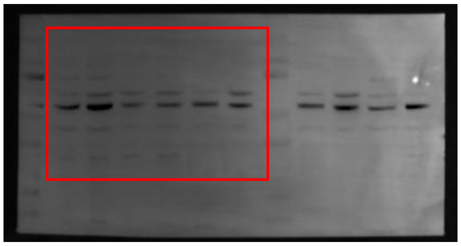

Fig4- a-tubulin (CD36)

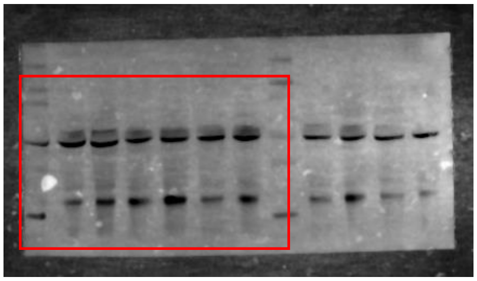

Fig5-NLRP3

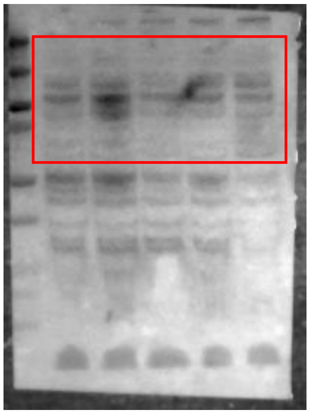

Fig5-cleaved- IL-1B

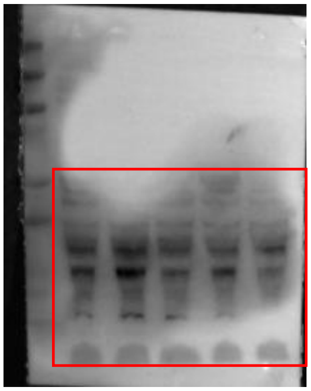

Fig5-a-tubulin (IL-1B/NLRP3

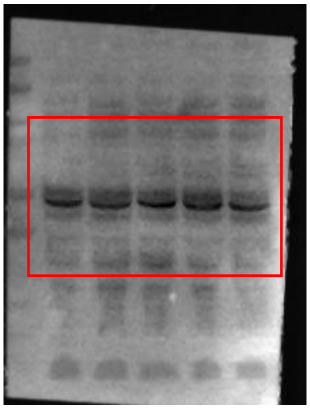

Fig5-ASC

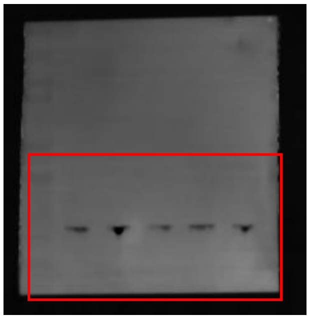

Fig5-a-tubulin (ASC)

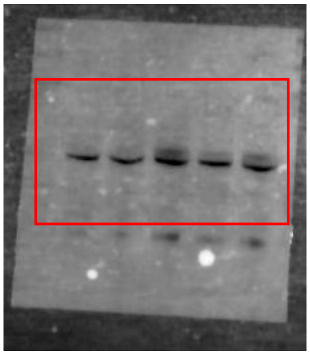

Fig5-cleaved-Caspase1

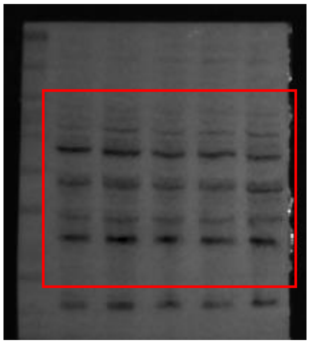

Fig5-a-tubulin (cleaved-Caspase1)

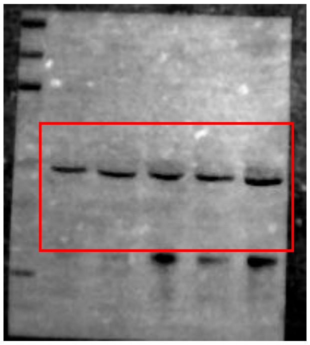

Fig6-CD36

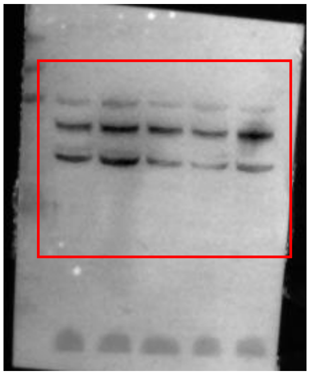

Fig6-a-tubulin (CD36)

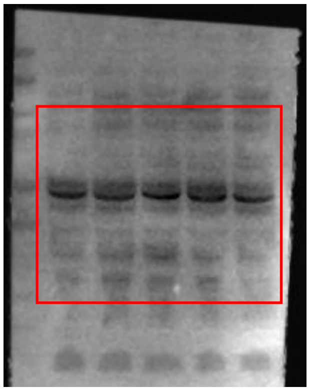

Fig5- KIM-1

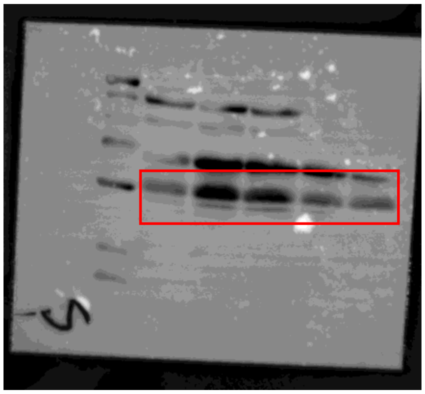

Fig5- NGAL

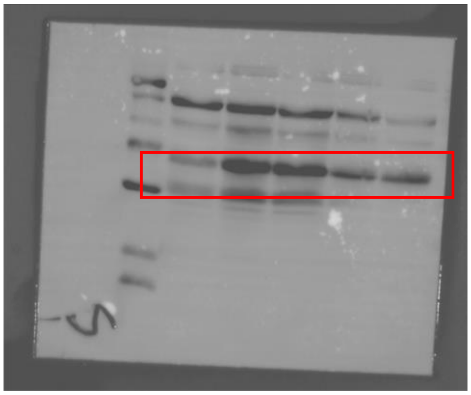

Fig5- L-FABP

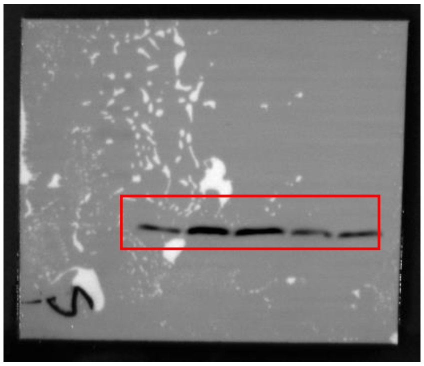

Fig5- GAPDH

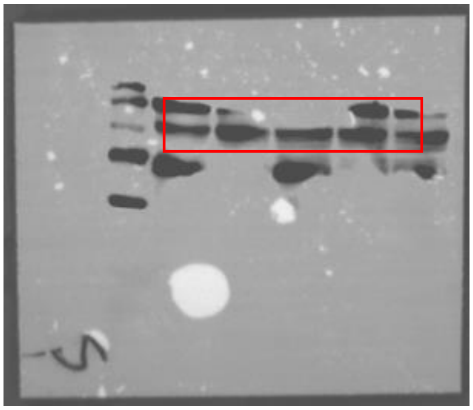

Fig5- C-GSDMD-D

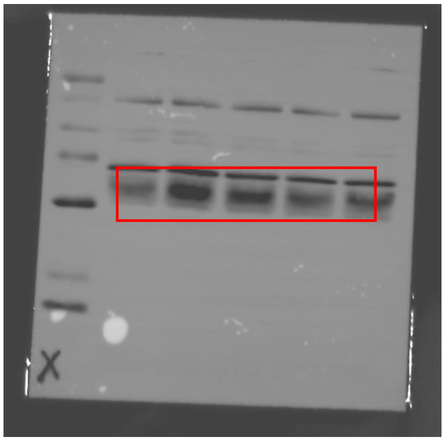

Fig5- GSDMD-D

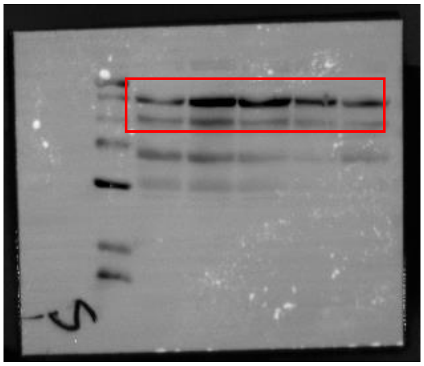

Fig7-cleaved- IL-1B

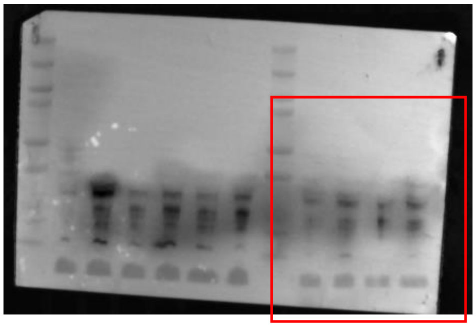

Fig7-NLRP3

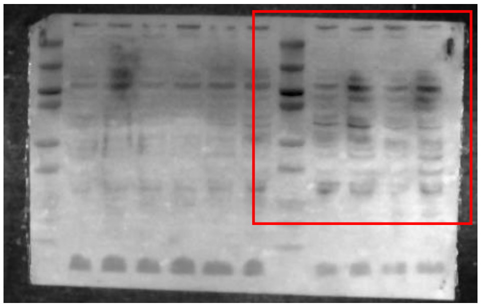

Fig7-a-tubulin (IL-1B/NLRP3

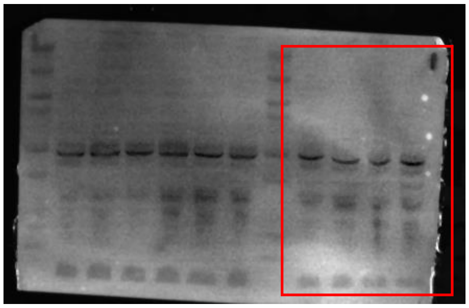

Fig7- ASC

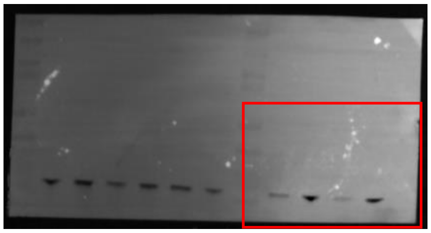

Fig7- a-tubulin (ASC)

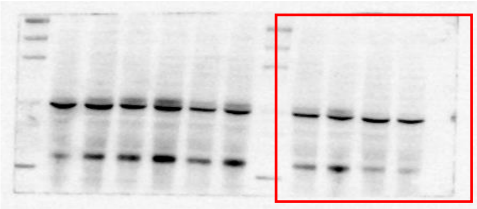

Fig7- cleaved-Caspase1

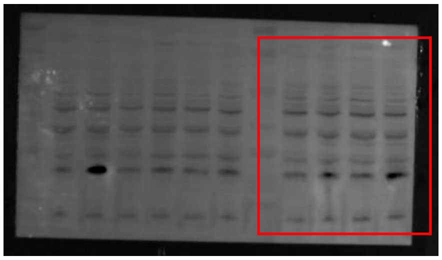

Fig7- a-tubulin (cleaved-Caspase1)

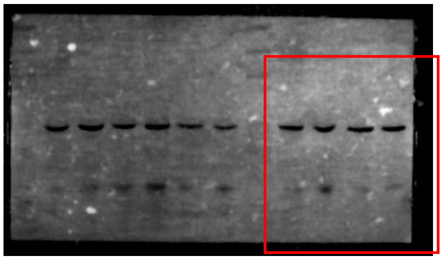

Fig7- CD36

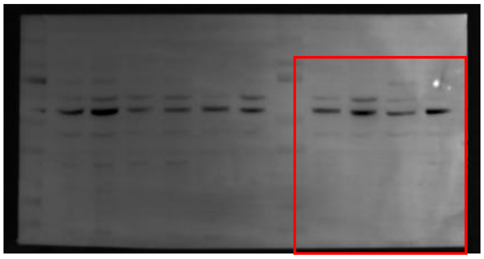

Fig7- a-tubulin (CD36)

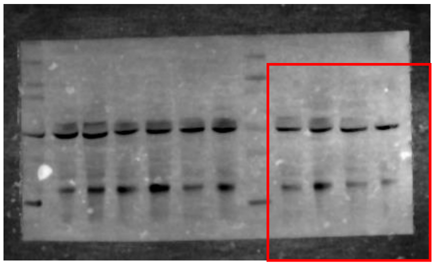

Fig7- KIM-1

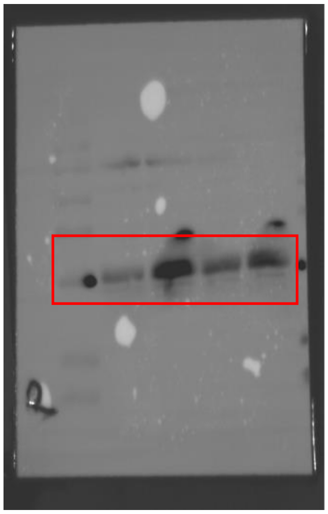

Fig7- L-FABP

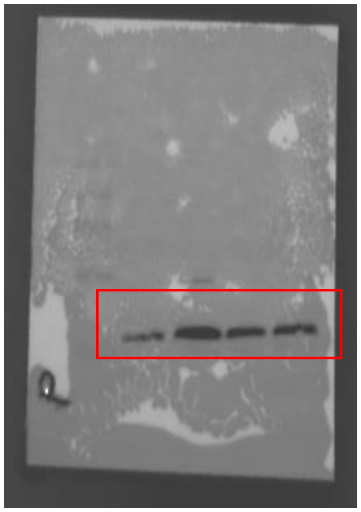

Fig3- a-tubulin

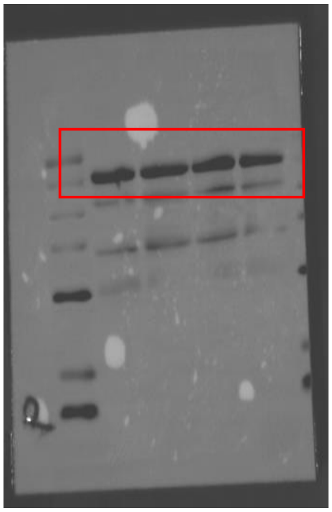

Fig7- NGAL

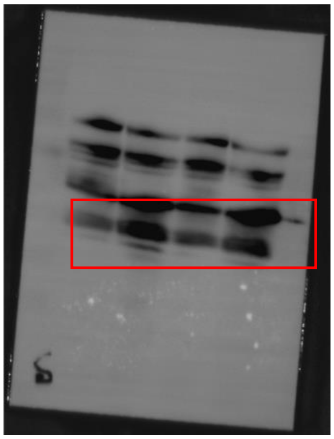

Fig7-C-GSDMD-D

Fig7-GSDMD-D

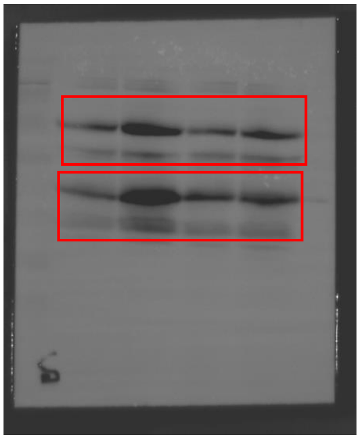

Fig7- GAPDH

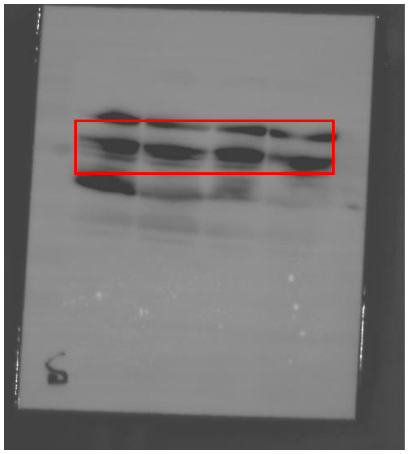

Supplement: Supplementary file 1 [file DataSheet1.pdf]
